# Temperature Influences Selective Mortality during the Early Life Stages of a Coral Reef Fish

**DOI:** 10.1371/journal.pone.0016814

**Published:** 2011-05-02

**Authors:** Tauna L. Rankin, Su Sponaugle

**Affiliations:** Marine Biology and Fisheries, Rosenstiel School of Marine and Atmospheric Science (RSMAS)/University of Miami, Miami, Florida, United States of America; Ecole Normale Supérieure de Lyon, France

## Abstract

For organisms with complex life cycles, processes occurring at the interface between life stages can disproportionately impact survival and population dynamics. Temperature is an important factor influencing growth in poikilotherms, and growth-related processes are frequently correlated with survival. We examined the influence of water temperature on growth-related early life history traits (ELHTs) and differential mortality during the transition from larval to early juvenile stage in sixteen monthly cohorts of bicolor damselfish *Stegastes partitus*, sampled on reefs of the upper Florida Keys, USA over 6 years. Otolith analysis of settlers and juveniles coupled with environmental data revealed that mean near-reef water temperature explained a significant proportion of variation in pelagic larval duration (PLD), early larval growth, size-at-settlement, and growth during early juvenile life. Among all cohorts, surviving juveniles were consistently larger at settlement, but grew more slowly during the first 6 d post-settlement. For the other ELHTs, selective mortality varied seasonally: during winter and spring months, survivors exhibited faster larval growth and shorter PLDs, whereas during warmer summer months, selection on PLD reversed and selection on larval growth became non-linear. Our results demonstrate that temperature not only shapes growth-related traits, but can also influence the direction and intensity of selective mortality.

## Introduction

Most animals have complex life cycles with multiple ontogenetic shifts, characterized by changes in morphology, physiology, behavior, and/or habitat [1]. This life history confounds our understanding of the processes affecting population dynamics, because pivotal events may occur during any stage or cumulatively across stages [1]. Metamorphosis from the larval to juvenile stage is thought to be a critical period characterized by a pronounced niche shift (e.g., from a pelagic to a benthic environment in marine organisms) and high rates of mortality [1], [2]. Early life history traits (ELHTs), such as larval growth rate or stage duration, often vary among individuals due to differences in genetic makeup, maternal contributions, and/or environmental influences. This trait variation coupled with high mortality rates create conditions for selective processes to occur [3]. Such “trait-mediated effects” can impact population dynamics and cascade through the trophic levels [4].

Temperature is well recognized in influencing metabolism and growth in poikilotherms [5]. Temperature differences in seasonal environments can increase variability of traits such as growth rate [6], size [6], [7], condition [8], and stage duration [9]. The consequences of such environmental influence can contribute to variation in survival [5], [10] and population replenishment of young [11], [12].

The growth-mortality hypothesis (GMH) [13] provides a theoretical framework for evaluating which traits may be selected for and whether consequences of performance based on larval traits propagate through to the juvenile stage (carry-over effects) [4], [14], [15]. It contends that faster growing individuals, that are larger-at-age, or advance more quickly to the next stage, will preferentially survive. Larval traits not only influence larval survival, but can also “carry-over” to influence performance and survival in subsequent stages [14], [15], [16], [17], [18]. Overall, larger size-at-hatching [19], [20], faster larval growth [15], shorter stage duration [21], and larger size- or higher condition-at-metamorphosis [22], [23], [24] generally enhance post-metamorphic fitness and survival. However, these trends in selective mortality are not pervasive. Patterns of selective mortality may not be maintained through time [25], [26] or occur in all populations of a species (e.g., *Thalassoma bifasciatum*) [15], [27]. Additionally, some field and laboratory studies have provided evidence that, contrary to the GMH, faster growing and/or larger individuals can be more vulnerable to predation [28], [29]. Thus, to maximize survival, behavioral or physiological trade-offs can result in less than maximally optimal traits [30], [31], [32].

Where environments are heterogeneous and variable, not only will the range of traits exhibited by poikilotherms vary, but the nature of their contributions to survival may change [33], [34], [35], [36], [37]. Extreme environmental conditions can be related to both increases in the selective nature of mortality [35] or randomization with regard to particular traits [33], [34]. Furthermore, environmental variables such as water temperature often covary with other factors such as food availability, predator composition and abundance, spawning output, and recruitment of young into the population, all of which can influence relative survival. Organisms associated with the coral reefs offshore of Florida, USA, are subjected to seasonal water temperature fluctuations of 10°C, yet populations of many taxa are replenished year-round. Contrasting seasonal processes may differentially influence the traits of surviving recruits during different seasons. We analyzed wild cohorts of a common reef fish settling to a subtropical reef system over a range of water temperatures to (1) examine how temperature influences variation in ELHTs and (2) test whether selective loss of particular variants within cohorts changes seasonally in relation to temperature.

## Results

After removing fish that were too old or did not settle during the appropriate 14 d settlement windows, 1357 of 1682 aged fish were used in the analysis (Table 1). Sixteen monthly cohorts spanned June 2000 to November 2005, with 13 tracked through time (i.e. sampled repeatedly), and divided into 4 age groups: settlement-stage larvae, newly settled recruits (1–7 d post-settlement), intermediate juveniles (8–14 d post-settlement), and survivors (15–21 d post-settlement) to examine patterns of selective mortality. The cohorts encountered mean water temperatures that ranged over 8.8°C, encompassing nearly all of the seasonal variability in this region.

**Table 1:** Sixteen cohorts of *Stegastes partitus* used in temperature analysis, 13 of which were used in selective mortality analysis (divided into age groups).

Cohort	Settle Month-Yr	Mean LT	Mean JT	Number of				Total N	LG (µm)	PLD (d)	Settlement Size (µm)	JG(µm)
				L	R	I	S					
5	Feb-03	20.6	21.2			18	18	36	7.8	29.2	234.3	4.6
6	Mar-03	21.0	21.3			14	14	28	7.8	29.9	239.1	4.7
9	Apr-05	22.9	23.9					14	8.0	29.6	244.1	4.8
8	Jan-04	23.9	23.4					19	7.8	30.2	228.6	
1	May-00	25.8	26.7		17	22	14	53	8.1	28.1	235.4	5.0
A	Jun-01	26.0	27.2	70	29	42		141	7.4	28.3	211.1	5.7
10	Oct-05	26.7	26.5					15	8.6	26.3	232.3	4.2
D	Jun-02	27.7	28.3	13	32	39		84	8.3	25.6	219.5	5.3
B	Jul-01	28.2	28.8	176	45	115	90	426	7.8	28.4	222.8	5.1
F	Jul-03	28.5	28.7	34	14	19		67	7.9	26.7	211.1	5.2
2	Jul-02	28.6	29.3		34	34		68	8.3	24.5	210.5	5.1
4	Oct-02	28.6	28.0			20	8	28	8.0	27.0	224.0	6.0
C	Aug-01	28.9	29.4	68	27	82	38	215	8.2	27.7	234.2	5.1
7	Aug-03	28.9	29.4			15	34	49	8.6	26.7	234.3	5.9
3	Sep-02	29.3	28.8			20	11	31	8.3	25.5	220.6	5.0
E	Aug-02	29.4	29.3	23	15	45		83	8.2	26.7	222.1	5.2

Mean LT and JT refer to near-reef water temperature (°C) averaged over the first hatch date to last settlement date (LT) and the first settlement date to last collection date (JT). Larvae (L) are settlement-stage larvae collected in light traps. Recruits (R) are juveniles from 1-7 d post-settlement; Intermediates (I), 8–14 d post-settlement; Survivors (S), 15–21 d post-settlement. Larval growth (LG) is mean otolith increment width over the entire pelagic larval duration (PLD); Juvenile growth (JG) is mean otolith increment width over the first 6 d post-settlement. Settlement size is otolith radius at the time of settlement.

**Figure 1:**
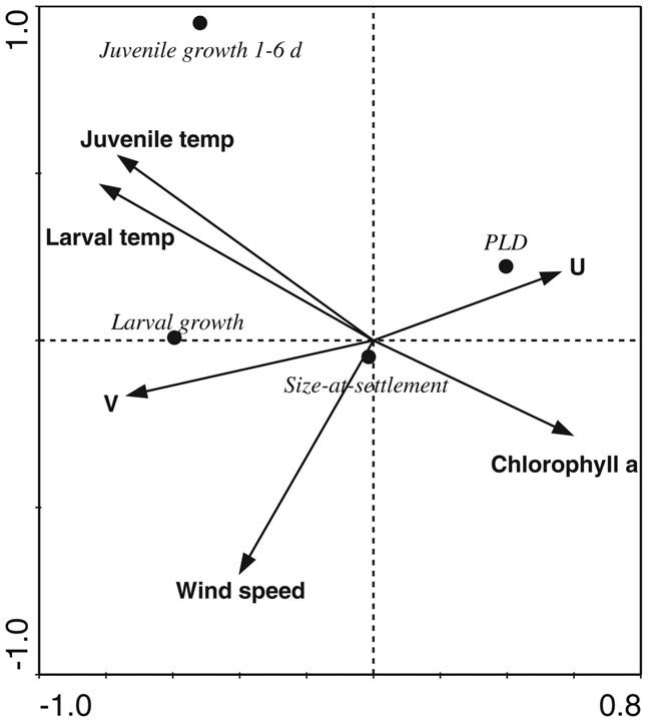
CCA ordination of *Stegastes partitus* early life history traits (mean larval growth, PLD, size-at-settlement, and mean juvenile growth during 0–5 d) and physical environmental factors (near-reef water temperature averaged over larval period and juvenile period, chlorophyll a concentration, wind speed, alongshore, and cross-self current velocity data).

The CCA for six environmental variables and four ELHTs yielded a significant biplot based on a global permutation test (1000 permutations; p<0.01; Fig. 1). The two axes explained ∼36% of the variation in the ELHTs. However, when tested using forward selection and 1000 Monte Carlo permutations, near-reef water temperature averaged over the larval period was the only environmental variable included in the model because the addition of any other variable did not significantly improve the fit.

**Figure 2:**
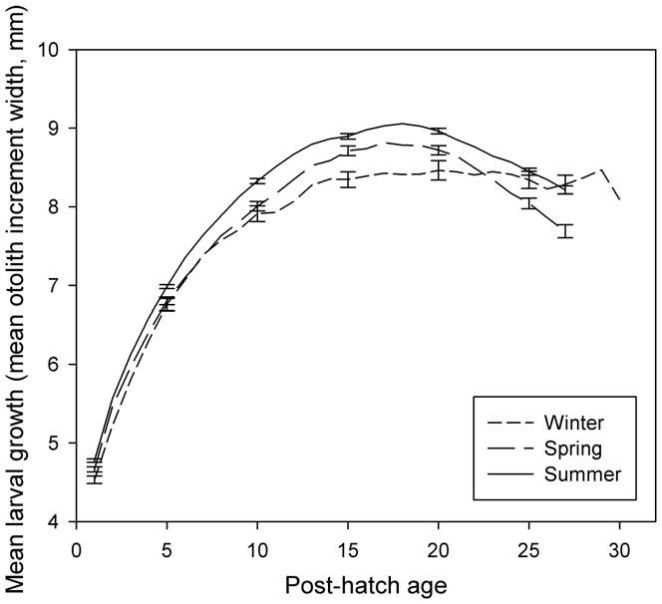
Mean daily otolith increment widths from hatching until settlement for 16 cohorts of *Stegastes partitus* settling during three different seasons. (A). Representative SE bars are provided at increments 1, 5, 10, 15, 20, 25, and 27.

The ELHTs examined varied among all 16 cohorts (Table 1) and many were significantly related to temperature. While the relationship between mean growth (otolith increment width) over the entire larval period and temperature was marginally non-significant (R^2^ = 0.24; p = 0.053), there was a significant positive relationship between early mean larval growth and mean water temperature (6–10 d: R^2^ = 0.40; p<0.01; Table 2). Likewise, size at 5, 10, 15, 20 and 25 d of age was positively correlated with temperature. However, the relationship between size-at-settlement and temperature was negative (R^2^ = 0.31; p<0.05), likely due to the strong negative relationship between PLD and temperature (R^2^ = 0.62, p<0.001), since size-at-settlement is a function of growth rate and the number of days spent in the plankton. Larvae grew rapidly in warmer water, but settled sooner, resulting in smaller sizes-at-settlement. Mean near-reef water temperature explained 38% of the variation in mean juvenile growth over days 1–6 (p<0.05). Seasonal larval growth trajectories demonstrated that summer *S. partitus* cohorts grew consistently faster than winter cohorts until day 27 of the larval period when most of the warmer-water fish were nearing settlement and either feeding less or devoting more energy to locating suitable settlement habitat, while cooler-water fish maintained more constant growth possibly because they were not competent to settle (i.e. they had longer PLDs/development times; RM-GLM: p<0.01; Fig. 2).

**Table 2:** Results of least-squares regressions between early life history traits and mean water temperature for 16 monthly cohorts of *Stegastes partitus*; *p<0.05, **p<0.01, ***p<0.001.

Trait		Relationship	R^2^
Larval growth	entire period	+	0.24
	1–5 d	+	0.23
	6–10 d	+	0.40**
	11–15 d	+	0.30*
	16–20 d	+	0.18
	21–15 d		0.00
	1–5 d prior to settlement		0.02
PLD		−	0.62***
Size-at-age	5 d	+	0.23
	10 d	+	0.37*
	15 d	+	0.45**
	20 d	+	0.44**
	25 d	+	0.31*
	settlement	−	0.31*
Juvenile growth	entire period	+	0.24
	1–6 d	+	0.38*

**Figure 3:**
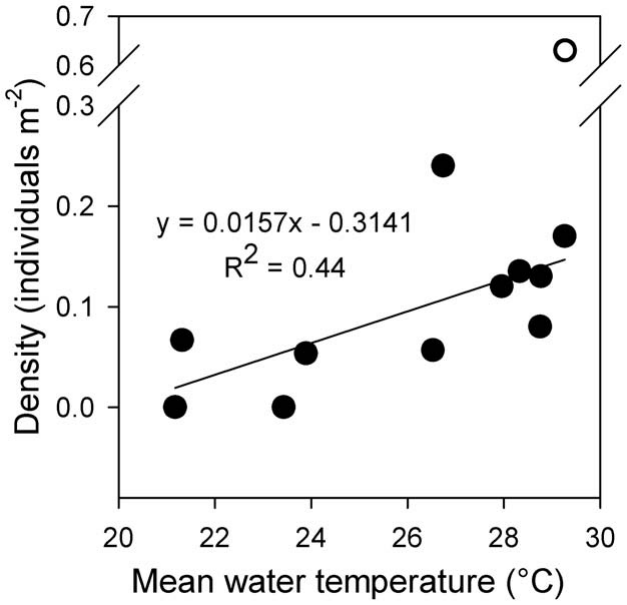
Relationship between new recruit density and mean water temperature over the juvenile period for all monthly cohorts of *Stegastes partitus* where recruit density was estimated. Relationship was significant when one outlier (o) was removed.

Density of new recruits varied from 0.00 to 0.63 recruits m^−2^ with a mean of 0.14 recruits m^−2^. There were no significant correlations between recruit density and any of the ELHTs. A positive relationship between recruitment magnitude and mean water temperature was significant once an outlier from the month with the largest recruitment event was removed (R^2^ = 0.44; p<0.05; Fig. 3).

**Figure 4:**
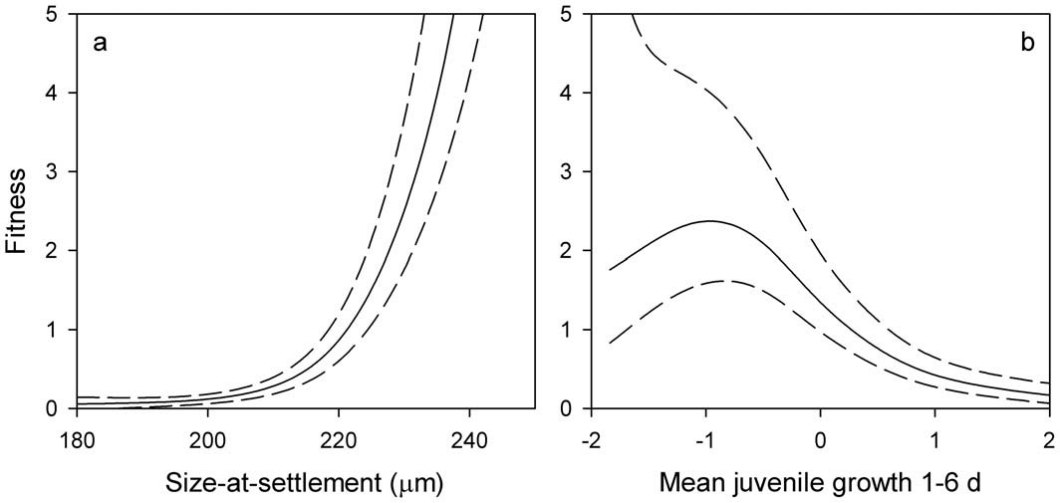
Intensity and shape (as cubic *b*-splines) of selective pressures acting on two otolith-derived traits, (a) mean size-at-settlement and (b) residuals of juvenile growth 1–6 d post-settlement regressed against seasonal water temperature, in 13 cohorts of *Stegastes partitus*.

In accordance with the GMH, individuals surviving up to three weeks on the reef were larger at the time of settlement than younger age groups (settlement-stage larvae, recruits, and intermediate age groups) regardless of temperature (Table 3), thus fitness increased with size-at-settlement (Fig. 4a). Contrary to the GMH, however, the survivor group also exhibited significantly slower growth over the first 6 d post-settlement than the intermediates or 6–7 d old recruits, once the extra variance due to differing water temperatures was controlled for (Table 4), reflecting consistently higher fitness at low juvenile growth (Fig. 4b).

**Table 3:** P-values of ANCOVA using *Stegastes partitus* otolith-derived traits (PLD, mean larval growth, size-at-settlement, mean juvenile growth rate over the first 6 d) as the dependent variables, age group as the factor, and mean water temperature over the larval period as the covariate.

Trait	Interaction	Temp	Age Groups	Tukey
Larval growth	<0.001			
PLD	<0.001			
Size-at-settlement	0.221	0.369	<0.001	L<R<I<S
Juvenile growth 1–6	0.962	<0.001	<0.001	R>I>S

Age groups: larvae (L) are settlement-stage larvae collected in light traps; recruits (R) are juveniles from 1–7 d post-settlement; intermediates (I) are 8–14 d post-settlement; and survivors (S) are 15–21 d post-settlement. Where interactions were non-significant, Tukey tests revealed where traits varied significantly among age groups.

**Table 4:** Results of GLM comparing *Stegastes partitus* otolith-derived traits (PLD and mean larval growth rate) among age groups (larvae, L; recruits, R; intermediate, I; survivors, S) for three different seasonal periods.

Cohorts	Trait	P-value	Tukey
Winter	Larval growth	0.014	I<S
	PLD	0.023	I>S
Spring	Larval growth	<0.001	L<R, I, S
	PLD	<0.001	L>R, I
Summer	Larval growth	0.276	
	LG 1–5	0.005	L>R
	LG 6–10	0.002	L>R, R<I
	LG 11–15	0.005	L>R
	LG 16–20	0.282	NS
	LG 21–25	<0.001	L<R<I<S
	LG Last 5 d	<0.001	L<R<I<S
	LG 6–10 d prior	0.057	NS
	PLD	<0.001	L<R, I<S

Where significant relationships occurred, Tukey tests revealed where traits varied significantly among age groups.

**Figure 5:**
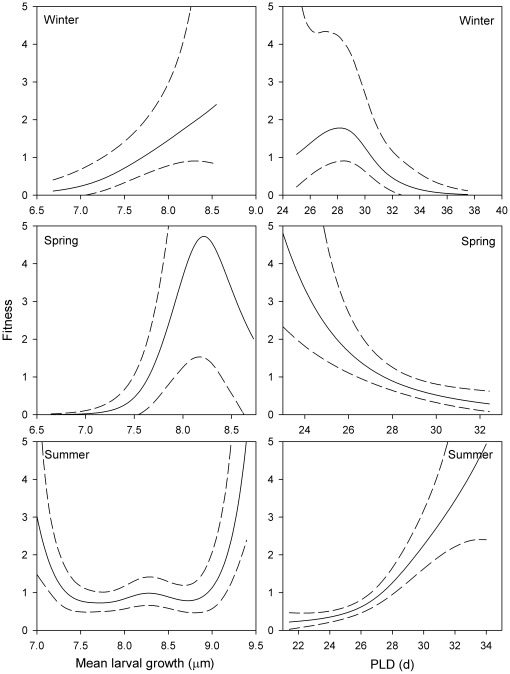
Intensity and shape (as cubic *b*-splines) of selective pressures acting on two otolith-derived traits, mean larval growth and PLD, in 13 cohorts of *Stegastes partitus* for three different seasonal periods, winter, spring, and summer.

Because temperature interacted with larval growth and PLD to result in seasonal differences in selective mortality (Table 3), the monthly cohorts were grouped by season and the two traits reexamined by age group using GLM. During the winter months, survivorship patterns followed the GMH: survivors (i.e. those with higher fitness) exhibited faster mean larval growth and had shorter PLDs than the intermediate group (Table 4, Fig. 5). These trends were more or less maintained during the warm spring months, but changed sharply during the warmest months. During summer, PLD-based selection reversed and individuals with shorter PLDs were selected against (Table 4, Fig. 5). The selective processes that acted on larval growth were more complex: There was no significant linear selective mortality evident in mean larval growth over the entire period (Table 4), likely due to selection against faster growth during the beginning of the larval period (Days 1–5, p<0.01; Table 4), but against slower growth towards the end of the phase (Days 20–25, p<0.001; Table 4). The increased fitness of the fastest and slowest growers, as indicated by the *b*-spline, points to the existence of disruptive selection (Fig. 5).

## Discussion

Our results demonstrate that not only does temperature influence the ELHTs that are important to survival during the critical periods of larval and early juvenile life, but temperature can also affect how selective mortality processes act on those traits. Temperature influences growth-related traits, including daily growth, size-at-age, and length of development, of a diversity of organisms [7], [18], [38], [39], [40] and mortality is frequently selective with regard to those traits [14], [15], [16], [20], [23], [25], [41]. However, there has been little evidence of a direct link between temperature and patterns of selective mortality [34], [35], [37]. Results of the present study demonstrate that seasonal changes in temperature not only influence the intensity of selective mortality, but can reverse the selective pressure on particular traits.

### Temperature mediated traits and recruitment

ELHTs varied among the 16 damselfish cohorts we examined and a significant portion of the variation could be explained by temperature. Mean near-reef water temperature explained 62% of variation in PLD: larvae in warmer water spent less time in the plankton. Additionally, warmer water fish settled at smaller sizes, largely because they experienced a shorter larval duration. While there was no significant relationship between overall larval growth and water temperature, early juvenile growth (averaged over the first 6 d) was faster during the summer compared to the winter (R^2^ = 0.38; p<0.05). Relationships between temperature and ELHTs in only larvae were similar, but more significant (accounting for 57% of variation in larval growth and 71% of variation in PLD), suggesting that selective mortality tended to dampen temperature-trait relationships (*unpubl. data*). Recruitment magnitude varied seasonally, but was unrelated to any of the ELHTs examined. Although recruitment of reef fishes to other locations, such as Panama, is known to be significantly related to larval growth or size-at-settlement [12], [42], the oceanographic setting of the two systems differ substantially. Stochastic physical processes (e.g., transient mesoscale eddies) prevalent in the Florida Keys (FK) likely disrupt fundamental biological relationships [43]. Recruit density did covary with water temperature, but the underlying mechanisms are unclear. Increased spawning output in warmer water is one possibility.

### Patterns in selective mortality

Regardless of water temperature, larger *S. partitus* settlers preferentially survived the first 2–3 weeks post-settlement. This is consistent with the ‘bigger-is-better’ component of the GMH and is evident in another pomacentrid, *Pomacentrus amboinensis*, on the Great Barrier Reef [23]. However, the opposite trend in selective mortality has been observed for other reef fishes [24], including *P. amboinensis* sampled in the same area in a different year [25].

Contrary to what is predicted by the GMH, *S. partitus* survivors experienced slower mean growth over the first 6 d post-settlement than the younger age groups. Very fast growth is associated with reduced fitness in other taxa [44], [45], [46], [47], [48] and *S. partitus* at Lee Stocking Island, where faster juvenile growth was correlated with reduced survivorship [26]. Several potential trade-offs with accelerated growth explain why slower growth could be beneficial. Physiologically, rapid growth may be attained at the expense of development, tissue maintenance and repair, and, for fish, swimming capabilities [31]. Compromise of these functions could result in reduced ability to escape predation. For instance, Sogard and Olla [32] found that sablefish undergoing higher compensatory growth exhibited lower critical swimming speeds than fish with average growth rates. Potential behavioral trade-offs of faster growth are increased foraging activity or selection of foraging habitats that expose a greater risk of predation. For example, experimental trials with Pacific halibut demonstrated that fast-growing juveniles exhibited increased swimming activity, relative to slower growers [49]. To sustain high growth rates, *S. partitus* must spend unsheltered time foraging in the water column, increasing their exposure to predation. Increased territorial defense by larger individuals also likely reduces energy available for growth (*unpubl. data*).

The consistent importance of size-at-settlement (across all seasons and years) to survival of juvenile *S. partitus* illustrates how events occurring in one stage can carry over to influence survival during subsequent stages. The extents to which these carry-over effects continue to influence fitness in the adults that survive to contribute to the population are unknown, but could have important consequences for population dynamics.

### Temperature induced changes in selective mortality

Patterns in selective mortality acting on larval growth and PLD changed with seasonal water temperature. In the cooler winter and spring months, individuals with slower larval growth rates and longer PLDs were selectively removed from the population, in accordance with the GMH. However, during the summer months, there was a reversal in selection to that against individuals that spent shorter periods in the plankton. There was no significant directional selective mortality acting on larval growth during the warmest months, and evidence of disruptive selection (i.e. loss of individuals with intermediate growth rates). Overall growth during the warmest months was faster (note shift in range in Fig. 5) than the other seasons and two different strategies appear to enhance survival during these months: fast larval growth or slower larval growth coupled with a longer PLD. Either extreme resulted in relatively large sizes-at-settlement, an advantage for juvenile survivorship. Although competency to settle is more likely related to a minimum settlement condition or stage of development than size (settlement-stage larvae vary in size), faster growers probably attain this more rapidly than slower growers [15].

Stabilizing selection appears to be operating on PLD over seasonal scales, whereby winter cohorts of *S. partitus* juveniles tend to have longer PLDs with the longest PLDs selectively removed from the population and the reverse pattern occurring in the summer. The result is a relatively fixed larval duration from year to year. Many coral reef fishes exhibit rather low variability in PLDs, which is thought to be limited by a minimum developmental period and the maximum extent that settlement can be delayed [50]. Developmental time may be influenced by seasonal changes in water temperature, but patterns of selective mortality of *S. partitus* recruits suggest that those that settle outside of the optimal range are less fit as juveniles.

Several recent studies have examined the influence of temperature on selective mortality [34], [35], [37]. One study of wild caught silver-stripe round herring *Spratelloides gracilis* detected size-selective and growth-selective mortality during the coolest month when their growth was the slowest, but not in other cohorts [35]. Grorud-Colvert and Sponaugle [37] similarly observed strong selective mortality in favor of *T. bifasciatum* of higher condition during the coldest months, but no consistent directional selection during the warmest months. Our study is one of the first to demonstrate that seasonal changes in water temperature can reverse the selective nature of mortality on particular traits, such as PLD. Thus, not only did temperature influence growth-related ELHTs, but also the intensity and direction of selective mortality that acted on those traits. What is optimal for survival during one season may not be so in others [33], [35]. This may explain why studies conducted over short periods of time have yielded contradictory results, and highlights the importance of research that encompasses seasonal variability. The relative contribution to the adult population from recruits arriving during the winter months versus those settling during the summer is unknown. Recruitment is lower in the winter, but due to potentially stronger selective mortality, winter survivors may be particularly vigorous and contribute disproportionately to the population.

Although temperature appears to play a pivotal role in early survival, it is difficult to disentangle the direct effects of temperature from other factors that vary seasonally, including predator and prey composition and abundances. Enhanced prey abundances can positively influence growth rates and recruitment success [51], while elevated predator abundances can exert greater selective mortality pressures on particular individuals [52]. We did not detect a strong relationship between growth-related traits and primary production (chlorophyll a concentration), but without measures of zooplankton prey and piscivore abundances, we cannot eliminate the interaction of these factors. Furthermore, intensity of selective mortality could be related to settlement density [53]. We estimated recruit density, which did not significantly covary with any of the ELHTs by age group. However, because recruitment was estimated after settlement, it is conceivable that initial settlement densities were important, but our ability to detect their relationship to selective mortality was obscured. While temperature is known to have a direct influence on the scope of growth-related traits in poikilotherms, seasonal variation in these other environmental factors are likely to also influence the degree to which certain traits are selected against.

Taking into account ongoing global climate change, our results suggest that patterns in selective mortality may diverge from what currently exists. For instance, for *S. partitus* that experienced warmer water temperatures, patterns in selective mortality reversed from selection against longer PLDs, to selection against shorter PLDs. While this suggests stabilizing selection over seasonal scales, in the context of long-term climate change, one of these seasonal patterns may diminish in importance (i.e. selection against longer PLDs during cooler months). This situation is further complicated by the fact that PLD was negatively correlated with temperature. Under warmer conditions, PLD should be shorter, but selective mortality against short PLDs could result in lower fitness of a large portion of the settling cohort. The larger implications are that adaptive trends could change with increasing global temperatures, leading to the evolution of new early life strategies.

## Materials and Methods

### Study site and physical data sampling

Population replenishment of organisms to the Florida Keys reef tract (FKRT) is influenced by the dominant Florida Current (FC) and the formation and propagation of mesoscale eddies along the FC front [43]. Mean water temperature along the FKRT varies seasonally by approximately 10°C and can significantly influence variation in ELHTs and recruitment strength of reef fishes [40]. Thus, to characterize the inshore environment of the FKRT, daily water temperature and current data were obtained from the National Underwater Research Center, which continuously records at 21 m depth at Conch Reef in the upper FK (24°59′N, 80°25′ W). Additionally, SST and ocean color images of the FC, provided by the University of South Florida's Institute for Marine Remote Sensing, were used to describe the offshore environment.

### Biological sampling

The bicolor damselfish *Stegastes partitus* is a zooplanktivorous fish common to the FKRT and greater Caribbean. Adults maintain benthic territories in which they spawn demersal eggs on a monthly basis, throughout the year [54]. Larvae spend a mean of 30 d in the plankton before settling to the reef [55] and metamorphosing into juveniles overnight. The timing of settlement appears to be synchronized with lunar phase, with pulses occurring during the third quarter and/or new moon [54], [55], [56]. Seasonal peaks in settlement to the FKRT typically occur during summer months [56]. *Stegastes partitus* is an ideal model species for studying processes affecting the early life history because they are common throughout their range, are integral to the trophic dynamics of the reef community, are easy to observe and collect at all life stages, and have otoliths (ear stones) that provide a daily record of events occurring during early life.

To capture settlement-stage *S. partitus* larvae as they settled to the reef, light traps were deployed intermittently from June 2001 until January 2004 in conjunction with two other studies [43], [56]. Three to six traps were deployed at night at a combination of four study sites located in the Florida Keys National Marine Sanctuary: French Reef (FR; 25°02.06′N, 80°21.00′ W), Sand Island Reef (SI; 25°01.09′N, 80°22.08′ W), Molasses Reef (MO; 25°00.74′N, 80°22.40′ W), and Pickles Reef (PI; 24°59.23′N, 80°24.88′ W). Larvae were used for analysis when sample sizes were sufficiently large (n≥20). Newly recruited *S. partitus* juveniles were censused and collected 3–5 d later at the same sites, with the exception of FR and MO, which are protected areas where benthic sampling was not permitted. Instead, recruits were collected just north of FR (NF; 25°02.53′N, 80°20.64′ W). Recruit density was estimated by counting the number of newly recruited juveniles within ∼20 haphazardly placed 5×1 m transects. Approximately 30 recruits were collected by divers using hand nets and the anesthetic Quinaldine. Subsequent collections were made every 3–5 d thereafter for approximately two weeks, resulting in a total of three or four collections for each cohort (or group of fish settling at the same time; see below). All collected juveniles were immediately stored in 95% EtOH following collection to preserve their otoliths.

### Otolith analysis

Standard procedures were used to digitally measure the standard length (SL) of each fish, dissect out and clear (in immersion oil) their otoliths, read and innumerate increments (at 400×) along the longest axis of the clearest lapilli, and validate readings (*sensu* Sponaugle and Pinkard) [57]. Otolith microstructure analysis was utilized to determine age (number of concentric increments), timing of hatching, timing of settlement, pelagic larval duration (PLD), larval and juvenile growth rates (increment widths), and size-at-age (otolith radius-at-each age, including settlement).

### Data Analysis

Standard length-otolith radius relationships (and relationships between residuals of both; [58]) were significant for each cohort, allowing us to use otolith measurements as proxies for growth and size. Relationships also did not differ significantly among the cohorts (*unpubl. data*), enabling inter-cohort comparisons. We used back-calculated dates of settlement to select only fish that settled within 14 d of each other for the analyses, resulting in 16 distinct cohorts. Utilizing the earliest hatch date and the earliest and latest settlement dates for each cohort, near-reef water temperature, chlorophyll a concentration, wind speed, alongshore and cross-shelf current velocity data, and all otolith-derived ELHTs (i.e. larval growth, PLD, size-at-settlement, juvenile growth) were averaged over the larval and juvenile periods separately.

Canonical correspondence analysis (CANOCO version 4.5) was utilized to distinguish the relative importance of the various environmental factors in influencing ELHTs. Because of the known importance of temperature in influencing growth, the relationships among ELHTs and water temperature were further examined by comparing mean cohort-specific water temperature with mean cohort-specific larval growth over the entire larval period and during separate 5 d intervals, PLD, size-at-age, and size-at-settlement using least-squares regression techniques (SYSTAT version 11.0). Likewise, mean cohort-specific juvenile growth over the first 6 d post-settlement was regressed against the cohort-specific water temperature averaged over the juvenile period.

Because ELHTs have been demonstrated to influence recruitment magnitude [12], [42], which in turn can affect growth, survival, and selective mortality [59], we examined the relationship between ELHTs and cohort density. Mean cohort-specific densities were regressed against the same mean cohort-specific ELHTs used in the regressions with temperature. Likewise, recruit densities were regressed against mean water temperature over the juvenile period.

Each cohort was further divided into four age groups: larvae, newly settled recruits (1–7 d post-settlement), intermediate juveniles (8–14 d post-settlement), and survivors (15–21 d post-settlement), which provided realistic timescales over which selective mortality could occur and resulted in sufficient sample sizes within the most age groups for each cohort. To determine whether mortality was selective with regard to any of the ELHTs, we compared the distribution of traits among age groups: A shift in the distribution of a trait between age groups would indicate directional selection. We used analysis of covariance (ANCOVA) to examine ELHTs among age groups with temperature as a covariate. For traits that could not be evaluated using ANCOVA (i.e. due to significant interactions between temperature and age group), monthly cohorts were divided into three seasonal groups by temperature (winter, spring, summer) and the traits were compared among age groups using general linear model (GLM) techniques. When significant differences were detected, Tukey's post-hoc test was used to identify significant pair-wise differences.

To determine whether growth trajectories of the different seasonal groups diverged through time, we examined daily growth by effect-coded season using repeated measures general linear model techniques (RM-GLM). To depict both linear and nonlinear forms of selective mortality, we estimated fitness functions using the cubic *b*-spline approach originated by Schluter [60] and adapted by Sinclair et al. [61] for two independent samples of a cohort, where *h(t)* is the probability that a fish with a particular value of trait *t* at an initial age was caught in a sample of survivors, given that it was collected in one of the two samples. As per Sinclair et al. [61], the initial group (larvae or new recruits) was coded as 1 and the survivor group as 3, and *h(t)* estimated using a generalized additive model assuming a binomial error distribution and a logit link:
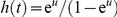
where u is a cubic *b*-spline smooth function of *t*, with a smoothing parameter λ chosen by generalized cross-validation.
